# Nutritional Physiology and Biochemistry of Dairy Cattle under the Influence of Heat Stress: Consequences and Opportunities

**DOI:** 10.3390/ani10050793

**Published:** 2020-05-03

**Authors:** Abdul Sammad, Ya Jing Wang, Saqib Umer, Hu Lirong, Imran Khan, Adnan Khan, Baseer Ahmad, Yachun Wang

**Affiliations:** 1 College of Animal Science and Technology, China Agricultural University, Beijing 100193, China; drabdulsammad1742@yahoo.com (A.S.); yajingwang@cau.edu.cn (Y.J.W.); 15121578@bjtu.edu.cn (H.L.); dr.adnan93@cau.edu.cn (A.K.); dr.baseerahmadkhan@gmail.com (B.A.); 2 Institute of Animal Sciences, Chinese Academy of Agricultural Sciences, Beijing 100193, China; saqibumar33@hotmail.com (S.U.); imran_talash76@yahoo.com (I.K.)

**Keywords:** heat stress, dairy cattle, negative energy balance, energetic metabolism, production, mitigation

## Abstract

**Simple Summary:**

Modern dairy cows have elevated internal heat loads caused by high milk production, and the effects of accumulating incremental heat are exacerbated when temperature and humidity increases in the surroundings. To shed this additional heat, cows initiate a variety of adaptive mechanisms including increased respiration rate, panting, sweating, reduced milk yield, vasodilatation, and decreased reproductive performance. Hormonal changes based on reciprocal alterations to the energetic metabolism are particularly accountable for reduced efficiency of the dairy production under the heat stress. As animals experience negative energy balance; glucose, which is also a precursor of milk lactose, becomes the preferential energy fuel. In the absence of proper mitigations, heat stress possesses potential risk of economic losses to dairy sector. Besides physical measures for the timely prediction of the actual heat stress coupled with its proper amelioration, nutritional mitigation strategies should target modulating energetic metabolism and rumen environment.

**Abstract:**

Higher milk yield and prolificacy of the modern dairy cattle requires high metabolism activities to support them. It causes high heat production by the body, which coupled with increasing environmental temperatures results in heat stress (HS). Production, health, and welfare of modern cattle are severely jeopardized due to their low adaptability to hot conditions. Animal activates a variety of physiological, endocrine, and behavioral mechanisms to cope with HS. Traditionally, decreased feed intake is considered as the major factor towards negative energy balance (NEBAL) leading to a decline in milk production. However, reciprocal changes related to insulin; glucose metabolism; failure of adipose mobilization; and skeletal muscle metabolism have appeared to be the major culprits behind HS specific NEBAL. There exists high insulin activity and glucose become preferential energy fuel. Physiological biochemistry of the heat stressed cows is characterized by low-fat reserves derived NEFA (non-esterified fatty acids) response, despite high energy demands. Besides these, physiological and gut-associated changes and poor feeding practices can further compromise the welfare and production of the heat-stressed cows. Better understanding of HS specific nutritional physiology and metabolic biochemistry of the dairy cattle will primarily help to devise practical interventions in this context. Proper assessment of the HS in cattle and thereby applying relevant cooling measures at dairy seems to be the basic mitigation approach. Score of the nutritional strategies be applied in the eve of HS should target supporting physiological responses of abatement and fulfilling the deficiencies possessed, such as water and minerals. Second line of abatement constitutes proper feeding, which could augment metabolic activities and synergizes energy support. The third line of supplemental supports should be directed towards modulating the metabolic (propionates, thiazolidinediones, dietary buffers, probiotics, and fermentates) and antioxidant responses (vitamins). Comprehensive understanding of the energetic metabolism dynamics under the impact of incremental heat load and complete outlook of pros and cons of the dietary ameliorating substances together with the discovery of the newer relevant supplementations constitutes the future avenues in this context.

## 1. Introduction

Modern dairy cattle back their origin to temperate climates. Intensive selection for high milk yield has made them less resilient to changing climatic conditions. Environmentally-induced hyperthermia in dairy cows leads to significant production losses. Noticeable sequels of heat stress (HS) are reduced feed intake and a variety of metabolic reshuffles, ending up in production and health losses. HS is an evident problem throughout the world [1]. HS negatively impacts a variety of performance parameters in dairy cattle including milk yield, growth, and reproduction and therefore possesses a significant financial burden of ~$900 million/year for the dairy sector in the U.S.A. alone [2]. HS adversely affects milk production and its composition in the dairy animals, especially in the animals of high genetic merit [3,4].

Investigating the biological mechanisms of HS behind lower animal production is crucial in devising mitigation strategies to ameliorate production declines. This prerequisite knowledge will help to suggest genetic, management, nutrition, and allied preventive mitigation strategies to secure and enhance dairy production. Over the last many years, advancement in cooling systems has lessened the production losses in hot weather conditions [5]. Modern dairy cattle are high milk yielders and energy status is the single most important factor in this context, a lot of studies have been conducted on understanding of the energetic metabolism and its repercussions under the influence of HS. However, a few advances have focused approaches to improve physiological and metabolic mechanisms in order to increase production of the heat-stressed dairy cattle. The objectives of this review are to summarize and highlight the physiological and metabolic acclimation of dairy cattle to HS, and to devise some impact mitigation strategies.

## 2. Heat Stress Assessment and Principals of Mitigation

This title provides up to date pre-requisite information of HS to the readership, global warming is expected to increase mean temperatures. When animal fails to lose radiant heat, which is mainly through convection, it suffers from the HS [6]. When ambient temperature exceeds 25 °C, cattle experiences HS [7]. Traditionally, temperature–humidity index (THI) is used to assess HS in dairy production [8,9,10]. THI calculations are based on dry (Tdb in °C) and wet bulb temperatures (Twb in °C)/relative humidity (RH in %). Different formulas are given as: THI =0.72(Tdb+Twb)+40.6 [11] and THI = (1.8×T+32)–(0.55–0.0055×RH)×(1.8×T–26) [12].

THI has been successfully employed to asses HS in the dairy cattle at various conditions of indoor or outdoor [13] different climate and production systems [14,15]. The common consensus about THI scale is the upper threshold THI, upon which the cow starts to experiences signs of hyperthermia [7,10]. This threshold has been reported variable upon different systems, generally started from 67 [16] and 72 [5,17], THI above these limits initiated hyperthermia derived discomfort [15], altered physiology [11], decreased feed intake [18], and decline in milk yield and composition [19]. Besides this, THI threshold may act variably for different physiological parameter, being lower for respiration rate and higher for rectal temperature [17]. Careful THI measurement [15] combined with physiological parameters of HS assessment [20] can be useful to predict real heat load on cattle. For THI measurements climate conditions should be obtained at cow level (ambient) to evaluate the heat stress conditions that dairy cows are actually exposed to [21]. There is a need for re-ranking across the THI scale according to farming systems and different climatic conditions [14]. Therefore, surroundings microclimatic conditions along with allied physiological parameters should be taken in account to accurately predict HS in cows [15]. Development of automatic monitoring techniques makes it possible to combine THI with other physiological indexes (i.e., body temperature and activity), helping to comprehensively evaluate HS in dairy cows [20]. Individual animal temperature monitoring is of vital importance in this context. Rectal temperature [22], deep body temperature measurements [23] like, vaginal temperature [24], skin implanted thermo-loggers [25], rumen temperature [26], infrared thermography [27], and milk temperature [18] are various methods used so far for thermal monitoring of cows. Likewise, the individual cow monitoring of panting score, studies have advised that wind speed and solar radiation should also be taken in account while assessing HS through THI scale [28]. These factors are well-known to have a significant influence on the magnitude of HS.

However, each aforementioned method has some advantages and drawbacks, for example, vaginal temperature is accurate, milk temperature monitoring is easy [18], and infrared thermography give best results on forehead and eye area [27]. Rapidly evolving temperature and activity monitoring technology produce big data, which can be affectively used for modeling to predict accurate HS [29,30,31] and at the same time thermo-tolerant animals can be identified for possible future breeding. Identification of thermo-tolerant cows based on the physiological exhibits [32], defining their phenotypes together with the integration of molecular techniques [33] can help to achieve thermo-tolerance breeding in the cattle.

There is a necessity of boosting convective heat shedding by cows through structural engineering of barns and forced air flow because heat loss decreases with high incoming air temperatures. Evaporative cooling is the alternative, requiring partial enclosing of the barn; however, a limitation factor could be the humidity in ambient air. A better alternative approach is forced ventilation coupled with surface soaking of animals. Animals wetting can be achieved through sprinklers, foggers, and misters according to a situation which varies. The forced evaporative cooling may be useful in various parts of the dairy, the holding area for milking, the feeding lane, and the rest area [6]. These approaches vary according to barn structure, animal density, farming practices, climatic conditions, and technological adaptations. Consultation with the relevant experts is necessary for the farmers in this context, so that suitable solutions are due provided.

## 3. Physiological and Behavioral Modifications of the Cattle

Homoeothermic animals (depending on their physiological state) have a thermo-neutral zone where energy expenditure to maintain the normal body temperature is minimal, constant, and independent of environmental temperature [34]. Initial responses to the HS are considered homeostatic mechanisms and include increased water intake, sweating and respiration rates, reduced heart rate and feed intake [35]. If exposure to the thermal load is increased, heat acclimation (if survivable) is achieved via processes of acclamatory homeostasis [35]. However, this acclamation may not remain homeostatic if HS is prolonged and thereby the animal will initiate homeorhetic mechanisms to dissipate incremental heat load and acclimatize to stress conditions [36]. Increased heat dissipation (primarily through evaporative heat loss), reduced feed intake and milk yield and increased water intake are the typical signs of homeostatic responses in response to the HS [37]. When the temperature of the hypothalamus is above thermo-neutral zone, the heat loss mechanisms, such as vasodilatation and sweating are activated [36]. Heat-stressed cows consume less feed and consequently ruminate less, and this results in decreased buffering agents (ruminating is the primary stimulant of saliva production) entering the rumen. In addition, because of the redistribution of blood flow to the periphery (in an attempt to enhance heat dissipation) and subsequent reduction in blood delivery to the gastrointestinal track, thus disturbing the digestion process. Cows in thermal neutral conditions typically consume 12 to 15 meals per day but decrease eating frequency to 3 to 5 meals per day during heat stress [11]. The decreased frequency is accompanied by larger meals, which could have gut health consequences.

High body temperature due to HS evokes a series of physiological responses. Excessive flow of energy (in the form of unabated heat) into the body, in addition to energy depletion required for lactation and growth [38], can lead to reduced reproductive efficiency [39], deteriorated living conditions, reduced welfare, and in extreme cases death [28], unless the animal can activate various adaptive mechanisms to increase the external net energy flow. Documented physiological coping strategies used by dairy cows include increased respiration rate; panting; and sweating; decreased feed intake; reduced milk yield, growth, and reproductive performance. Cattle modify their feeding and drinking behavior; take feed in cooler hours, and frequent water intake.

When ambient temperature increases, cattle significantly increase heat production [40], therefore enhanced energy expenditure during HS is believed to originate from high physical adaptive activities like panting and sweating [41]. HS maintenance costs in lactating dairy cattle are estimated to increase by as much as 25% to 30% during heat stress [10,42]. However, due to a variety of acclamatory responses and depending on the severity and intensity of the HS, it will vary significantly. An increase in environmental temperature has a direct negative effect on the appetite center of the hypothalamus to decrease feed intake [10]. Chronic hyperthermia leading to severe or prolonged inappetence is also reported [11]. In summary, physiological responses and coping strategies under the influence of HS are surely posing extreme burden on dairy cows, which are mainly initiated and coordinated through autonomous nervous system [36]. High milk yield burden coupled with deteriorating livability principally needs adequate cooling and better feeding practices with high energy density so that a cow can withstand HS, successfully dissipate it, and at the same time maintain milk yield demands.

## 4. Hypothalamus–Pituitary–Adrenal Axis and Cellular Level Changes

Adjusting towards high thermal stress involves dynamic changes in the behavior and physiology, including hormonal changes in the hypothalamus–pituitary–adrenal (HPA) axis, releasing glucocorticoids and aldosterone [43]. Cortisol secretion tends to increase during the HS [44]. HS also causes an increase in circulating norepinephrine and epinephrine hormones [45]. These endocrinal alterations lower the plasma levels of Thyroxine (T4) and Triiodothyronine (T3), leading to low basal metabolic rate and thus decreased heat production [46]. Low circulatory levels of somatotrophins and high levels of IGF-II significantly alter the dietary intake of HS animals [47]. During HS there is a reduction of hepatic GH receptor abundance, which may be involved in the GH-dependent liver process like regulation of gluconeogenesis [48]. A study showed high levels of IGF-1 in high producing lactating cows under the influence of HS, indicating high hepatic triglycerides clearing [49]. Contrastingly, another study suggested that HS can decrease *IGF-1* mRNA abundance, which could be related to alterations in gluconeogenesis pattern [50]. Decline in T3 and T4 is the immediate adaptive response [51], which along with decreased plasma GH has a synergistic effect to reduce heat production [52].

**Figure 1:**
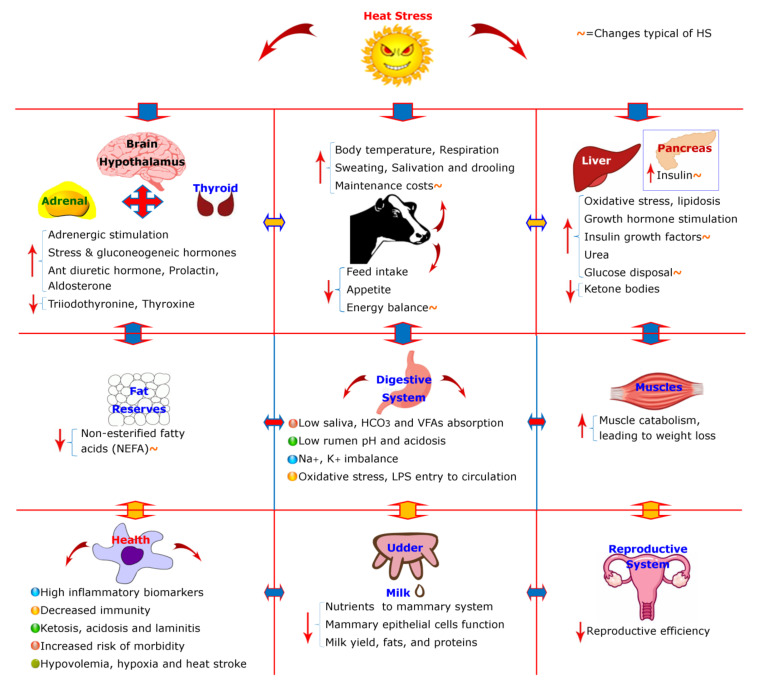
Summary of various heat stress-related physiological and biochemical changes occurring at various systems level in the body of the dairy cows. Phrases with the indication of “~” indicate changes typical of heat stressed animals. (LPS=lipopolysaccharides, VFAs= volatile fatty acids).

During chronic HS, aldosterone level decreases due to excessive loss of K+ in sweating. In cattle, heat exposure and dehydration during HS resulted in a sharp increase in plasma ADH concentration which was associated with a significant decrease in urine output [53]. An increase in prolactin (PRL) concentration is reported; being a homeorhetic hormone it may help in the adaptive metabolic responses in HS. A study proposed that elevated PRL is involved in meeting increased water and electrolyte demands of the heat stressed cows [54]. Interestingly, a review summarized that prolactin together with other involvements, supports insulinemia typical of the HS [55]. Alteration in the endocrine mechanisms is having a negative effect on the appetite center of the hypothalamus during the HS [56]. Besides these, heat shock proteins (HSPs) are the typical molecules of hyperthermia involved in cell protection while HSP70 has a major role. HSP70 expression magnitude is a better predictor of the HS [57,58]. HSPs have shown to improve insulin sensitivity [59], suggesting its role to improve animal productivity under HS. Figure 1 summarizes the physiological and biochemical alterations occurring in response to the HS. In summary, the initial response to HS is a homeostatic response driven by the autonomic nervous system, and if HS persists for relatively longer durations, acclimatization and seasonal adaptability is achieved through the endocrine system and homeorhetic mechanisms in the body [36].

## 5. Negative Energy Balance (NEBAL) in Lactating Cows

Lactation itself exerts a magnitude of stress on the cattle and a significant proportion of feed derived nutrients are partitioned towards milk synthesis. Modern dairy cows experience negative energy balance (NEBAL) during early lactation, in order to support high lactation demands. There are significant changes in energetic metabolism to ensure the supply of feed and tissue-derived nutrients to the mammary system. Due to the reduced feed intake and the proposed increase in maintenance costs, the HS cows decrease milk yield but still experience NEBAL [60]. High non-esterified fatty acid (NEFA) and low glucose levels are typical in early lactation cows [61]. NEBAL reduces insulin concentration and sensitivity, thus having a lipolytic effect. It stimulates NEFA export from adipose tissues/lipolysis in response to catecholamines while simultaneously inhibits classical insulin activity [62]. Lipolysis response spares glucose from primary homeorhetic responses and instead is directed towards lactation support [63,64,65]. During thermo-neutral NEBAL insulin tends to increase growth hormone (GH) receptors in liver and adipose tissues [66]. Circulating NEFA and derived ketone bodies help to overcome the energy and lactation requirements of the cows in NEBAL. These alterations are mainly mediated by somatotrophins (ST) increase [65]. This phenomenon better explains the weight loss of cows in early lactation. Peri-parturient NEBAL can lead to increased incidence of metabolic disorders [67]; facilitating the cow to adapt successfully through appropriate nutrition and management is a key to maintain cow health and farm economics [68].

## 6. Negative Energy Balance (NEBAL) Typical to Heat Stress

Reduced feed intake caused by HS is thought to be a primary response towards decreased milk yield [41,54]. However, now it is known that reduced feed intake is merely responsible for about 35% of the HS induced drop in milk production [69]. Rather major effects of HS consequences are intake-independent changes in nutrients partitioning. Another study held 50% of HS induced feed intake reduction being responsible for lower lactation yield. Additional reduction causes are born by intake independent changes in post-absorptive glucose and lipid metabolism [4]. This brief introduction makes it clear that indeed decreased intake is responsible for lower production, however metabolic alterations during HS drive the additional stress and decreased milk yield. Consequences of this metabolism shift are extended beyond the production; reproduction and health are also affected. Below we will discuss the individual components of this context.

### 6.1. Insulin and Glucose Axis of Heat Stress

The variety of post-absorptive metabolic changes occurring in HS cow, notable ones are high insulin activity, failure of adipose tissue mobilization and thus failure to enlist glucose sparing mechanisms [4,69]. These changes lead to production losses to an extent which is more than that for cows with poor nutrition status. These causes and effects are also shown to be similar for growth parameters, with major part be explained by the HS-induced reduction of feed intake [70]. HS cows are shown to have increased basal insulin concentration and high insulin response to a glucose tolerance test [4,42]. Exact HS specific insulin increase has been long debated, but it appears to be adaptive and protective in nature towards stressors [55]. Contrastingly a review summarized that prolactin together with other involvements; support insulinemeia typical of HS, this high insulin concentration can also be contributed towards many other things, like, lipopolysaccharides (LPS), and high concentration of intracellular Ca+ [55]. HS may alter glucose uptake in many ways, it can be tissue-specific, how much part insulin-independent glucose disposal accounts during HS; needs to be investigated thoroughly, as insulin-independent glucose transporters (GLUTs) also tend to increase during in-vitro HS [71]. Conversely, when heat-stressed and cooled cows were compared in a study, cooled cows have low glucose levels, low insulin response and increased fats metabolism with relatively high milk yield [72].

### 6.2. Insulin and Lipids Metabolism Axis of Heat Stress

Early lactating cows and those with underfed proper diet tends to mobilize fat reserves to keep up with high energy demand of lactation [73]. Decreased nutrient intake being an utmost indicator of the HS; is generally associated with NEBAL [4], bodyweight loss [69], and elevated NEFA levels [74,75]. HS causes a marked increase in circulating cortisol, norepinephrine and epinephrine levels [45], catabolic signals that normally stimulate lipolysis and adipose mobilization. But this is not the case in HS dairy cows; instead, NEFA levels go down significantly [4,55]. An experimental study showed that HS cattle have blunted NEFA response towards epinephrine challenge [69]. Circulating NEFA and derived ketone bodies helps overcome NABAL, and this lipolysis response spares glucose from primary homeorhetic responses and instead is directed towards the lactation support [64,68]. There is high insulin activity during HS as described above. Insulin is also a potent anti-lipolytic hormone [62] and may explain why heat-stressed animals do not mobilize adipose tissue triglycerides. Instead of NEFA mobilization, HS has been shown to increase lipoprotein lipase, suggesting anabolism of triglycerides. Limiting adipose tissue mobilization is the key step by which heat stressed animals are prevented from employing glucose-sparing mechanisms normally enlisted to maintain milk or skeletal muscle synthesis during periods of temporary malnutrition. The lack of available NEFA to systemic tissues for oxidative purposes is coupled with the decrease of volatile fatty acids (VFAs) availability; leaving glucose and amino acids (AA) as the available oxidative substrates. Therefore, glucose is consumed as main oxidative fuel in the HS animals [76]. Now it is clear that the HS poses a sever burden on the energetic metabolic balance of the dairy cow. Glucose as preferential fuel could debilitate animal, compromise homeostatic physiological responses of heat abatement and decrease production and reproduction potential of the cows. Therefore mitigation strategies, whether physical or nutritional, should be focused on improving energetic metabolism through maximizing glucose rescue.

### 6.3. Protein Metabolism in Heat Stress

Amino acids of blood are known to synthesize the major components of milk protein in bovine mammary glands. Many studies indicate marked changes in circulating amino acids under catabolic conditions [77] and HS [78], because of the insulin resistance in peripheral tissues and use of AA in gluconeogenesis [79]. HS reduces milk protein content, and changes the AA profile of dairy cows, suggesting that more AA are required for maintenance (immune response and gluconeogenesis) but not for milk protein synthesis under HS [78]. Highly significant variation of Hb, PCV, plasma glucose, total protein, and albumin has been reported for the different temperature exposure [80]. Muscle anabolic and nucleic acids synthesis is also shown to be severely hampered by HS [81].

In addition to adipose tissue, skeletal muscle is also mobilized during periods of inadequate nutrient intake (in thermal neutral conditions) to support lactation. Heat-stressed cows [82] have increased plasma urea nitrogen levels. A better circulating indicator of muscle catabolism is either 3-methyl-histidine or creatine, both of which are increased in heat-stressed lactating cows [83]. HS has been reported to interfere with nitrogen metabolism and cause nitrogenous repartition in dairy cow and decreases milk protein content while increasing milk urea concentration [84]. Additional evidence suggests that HS alters protein metabolism and milk protein levels decreased in heat-stressed cows [69,82].

The increase in skeletal muscle protein catabolism is interesting as the role of insulin is to promote protein anabolism [85]. This change in function can be attributed to various modifications in energetic metabolisms at multi-levels. HS can increase cell membrane permeability causing Ca+ leakage that could increase protein sensitivity to HS [86]. The concentration of blood alanine, glucose, aspartate, and glycine, associated with gluconeogenesis, are shown to be significantly increased under HS [78]. Conclusively we can say that skeletal muscle catabolism may be a strategy to support gluconeogenesis [87], rather than for oxidation purposes because the efficiency of capturing ATP from amino acid oxidation is low. Oxidative stress [78], immune response and unique gluconeogenesis [55] depleted a large number of amino acids, thus decreased the availability of amino acids for milk protein synthesis in our study. Therefore, high extra-mammary protein catabolism and amino acid consumption during HS have been accounted for low milk proteins and milk yield [88].

## 7. Effects on Milk Production

Continual genetic selection for greater performance results in increased HS sensitivity and a resulted in a decreasing trend in the lactation curve as well as poor milk quality in dairy animals during summer seasons. HS adversely affects milk production and its composition in dairy animals, especially animals of high genetic merit [3,4]. The components of milk are strongly affected by HS [89]. The greater number of somatic cells counted in milk during summer also shows that the hyperthermic environment severely affects the quality of the milk [90]. Furthermore, it is found that a hyperthermal environment could also reduce the milk protein content via the reduction of casein concentration [91]. Highly producing cows have been shown to utilize majority of its glucose in mammary tissues for milk production [92]. Due to high energy demand under HS, existing energy intake would not be enough to cover the daily requirements for the milk production. Total average milk production per cow was significantly (*p* < 0.05) higher in the spring period (42.74 ± 4.98 L) compared to summer (39.60 ± 5.09 L) [93]. HS above critical threshold decreases DMI by 9.6% and milk production by 21%, together with lower milk fat and milk protein in the summer season [3,94]. Reduced nutrient intake (indirect effects of heat) accounted for only about 35% of the heat stress-induced decrease in milk synthesis [69]. Additionally, the analysis of milk protein fractions also showed a reduction in percentages of casein, lactalbumin, immunoglobulin G (IgG) and IgA; 80% of these changes were associated with loss of productivity and 20% with health issues which might be due to disruption of internal homeostasis mechanism [95]. Similarly, lipid composition of milk is also disturbed during the HS [96]. Milk levels before HS, lactation stages, and parity are positively related to the extent of milk yield decline during HS. Studies have shown 35% decline for mid-lactation [69] and 14% for early lactation cows [94]. Besides milk yield and composition HS increased the somatic cell count of the milk [97] through initiation of immune response in the mammary tissue [98]. HS tend to activate immune system, which is energy intensive phenomena; therefore, the glucose consumption in dairy cow is increased [99]. Milk yield, composition, and quality are affected by HS. Failure to rescue milk yield due to shifts in energy metabolism; protein catabolism; alterations in lipid metabolism due to endocrine alterations; and immune response due to oxidative stress and inflammation are the major factors in this context.

## 8. Mechanisms Underlying Lactation Changes

Lactation is energy intensive process leading to high metabolic heat production. Which coupled with high THI load significantly jeopardizes animal welfare. Adjusting to this stress dairy animals decrease milk yield in order to produce less bodily heat. It involves direct and indirect responses at various physiological, endocrine, and biochemical modifications. For example, a study has shown that a derivative of b-casein acts like a ligand and binds mammary epithelial cells, disrupting potassium channels and ultimately reduces milk synthesis [100]. It shows that mammary epithelial cells are incapable of utilizing blood-derived milk precursors. Many studies have investigated these effects, showing that reduced feed intake accounts for only approximately 35%–50% of the decline in milk yield during HS [4,69]. Moreover, this is because of the disturbance of nutrient intake and milk production relationship during HS [101]. We have earlier discussed that how the NEBAL during HS differs from the normal; having high insulin activity, high glucose disposal, decreased NEFA mobilization, and high skeletal muscle metabolism. These changes are the significant consequences contributing to lower milk production.

ST and IGF-1 are the two most important lactation promoting hormones [62]. Normally, ST partitions nutrients toward the mammary gland by decreasing nutrient uptake by extra-mammary tissues and stimulating hepatic IGF-1 synthesis and secretion. Chronic heat-stressed cows (which are presumably in NEBAL) have reduced ST concentrations [47,69]. Consistent with reduced ST signaling through *STAT-5*, hepatic *IGF-1* mRNA abundance was less in heat-stressed animals [50]. Thus, the reduced hepatic ST responsiveness observed during heat stress appears to involve mechanisms independent of reduced feed intake. This physiological phenomenon may alter gluconeogenesis. Therefore, reduced IGF-1 may be one mechanism by which the liver and mammary tissues contribute towards a reduction in milk synthesis and increase nutrients utilization to maintain homoeothermic condition.

Furthermore, because of the reduction of feed intake and the rise of maintenance requirements, heat-stressed cows decrease the nutrient availability to mammary system [102]. Mammary gland of the dairy cattle requires glucose for milk lactose synthesis; which determine milk yield. However, to generate less metabolic heat during the eve of HS, the body tends to utilize glucose at a higher rate. Subsequently, the mammary system may not receive enough amounts of glucose for lactose synthesis leading to a decline in the milk yield. It could be the primary mechanism indicative of lower milk yield beyond for that of low fed cattle. HS has been reported to interfere with nitrogen metabolism and cause nitrogenous repartition in dairy cow and decreases milk protein content while increasing milk urea concentration [84]. Decreased milk protein levels from the heat-stressed cows [69,82] show that alpha- and b-casein synthesis is the most susceptible [103]. It is suggested that amino acids may be less used to synthesize milk protein under heat stress, but rather involved in immune response and gluconeogenesis [78]. The concentration of blood lysine that promotes milk protein synthesis was shown to be significantly decreased under the HS [78]. Conclusively, direct physical damage to mammary tissue; alteration in mammary gland ability to function normally; shift in energetic metabolism pattern; alterations to lactogenic hormones expression; and increased protein catabolism are the major underlying mechanisms of lactation changes during HS.

## 9. Rumen and Gut-Associated Changes

High loads of heat in the body of high producing cows during summer stress severely alters the feeding-drinking behavior, digestibility, gut health, and nutrients movement across the blood–intestine barrier. HS increases maintenance requirements of the dairy cattle [46]. The enhanced energy expenditure during heat stress is believed to originate from high physical adaptive activities like panting and sweating [41]. HS maintenance costs in lactating dairy cattle are estimated to increase by as much as 25% to 30% during heat stress [42,85]. However, due to a variety of acclamatory responses and depending on the severity and intensity of the HS, it will vary significantly. An increase in environmental temperature has a direct negative effect on the appetite center of the hypothalamus to decrease the feed intake [56]. Heat-stressed cows consume less feed and consequently ruminate less, and this results in decreased buffering agents (ruminating is the primary stimulant of saliva production) entering the rumen. In addition, because of the redistribution of blood flow to the periphery (in an attempt to enhance heat dissipation) and subsequent reduction in blood delivery to the gastrointestinal track, digestion-end products (i.e., volatile fatty acids (VFAs) are absorbed less efficiently leading to increased total rumen VFA content (and thus pH decreases). Furthermore, increased respiration rates also contribute to rumen acidosis because panting causes enhanced CO_2_ to be exhaled. In order to be an effective blood–pH buffering system, the body needs to maintain a 20:1 HCO_3_ (bicarbonate) to CO_2_ ratio. Because of the hyperventilation-induced decrease in blood CO2, the kidney secretes HCO_3_ to maintain this ratio. This reduces the amount of HCO_3_ that can be used (via saliva) to buffer and maintain a healthy rumen pH. Besides, panting cows often drool reducing the quantity of saliva available for the rumen. The reductions in saliva HCO_3_ content and the decreased amount of saliva entering the rumen make the heat-stressed cow much more susceptible to subclinical and acute rumen acidosis [11]. Cows in thermal neutral conditions typically consume 12 to 15 meals per day but decrease eating frequency to 3 to 5 meals per day during HS. The decreased frequency is accompanied by larger meals and thus more acid production post-eating. Chronic hyperthermia causing severe or prolonged in-appetence could also lead to subclinical and acute rumen acidosis [11]. As the nutritional mitigation strategies, proposed elsewhere and in this paper, to overcome adverse effects of HS on milk production involve energy-rich diets or supplementations improving energetic metabolism, risk of acidosis is a serious concern. Maintaining healthy rumen is of utmost importance for any type of nutritional interventions to overcome HS effects.

## 10. Health Consequences of Heat Stress

Health problems in heat-stressed ruminants are the consequence of nutritional and metabolic acclimation. A series of studies have described a higher risk of mortality during the summer months [104,105], and an increased death rate during heat waves [106]. In particular, due to increased maintenance requirements for thermoregulation and lower feed intake, summer transition dairy cows are more likely to experience subclinical or clinical ketosis [107] and are under higher risk of liver lipidosis [108]. Increased liver lipidosis probably compromises liver function and it is reported that heat-stressed cattle have reduced albumin secretion and liver enzyme activities [109]. Evidence is there that HS cause oxidative stress in the transition dairy cows [103]. Chronic hyperthermia also causes severe or prolonged in-appetence finally, resulting in subclinical and acute rumen acidosis [11]. The combination of a “hotter” ration and the cow’s reduced ability to neutralize the rumen directly increases the risk of rumen acidosis and indirectly enhances the risk of developing adverse side effects of an unhealthy rumen environment (i.e., laminitis and milk fat depression).

Acclamatory response of blood flow redistribution towards periphery from viscera under the influence of HS [110] can lead to intestinal hypoxia and oxidative stress [111,112]. These changes contribute towards loosening of the tight junctions, and thus, intestinal barrier impairment leads to entry of LPS in blood [110,112,113]. Endotoxemia has been reported in humans during heat stroke [114]. Interestingly, it has been found that LPS/endotoxemia in cows exhibited higher insulin circulation [115,116] which is characteristic of HS, as we discussed earlier. This insulin resistance in case of endotoxemia and its relationship with gut microbiota profile is well understood phenomena [117]. LPS-induced inflammation can activate the immune response; an energy-intensive process [118], in which cattle need more glucose, and therefore the process fail to rescue milk synthesis [99]. Feed suppression and inflammatory biomarkers are indicative of leaky gut, which increases in cows during HS. Therefore, leaky gut and the resulting LPS could markedly alter nutrient partitioning.

An association between severe heat stress and altered lymphocyte function was observed under field conditions [119]. Lacetera et al. [119] hypothesized that depressed cell-mediated immunity and an enhanced humoral response might be related to heat-induced increases in circulating cortisol by causing a shift from a T-helper 1 (Th1; cellular) to a Th2 (humoral) pattern of immunity leading to increased infection susceptibility. Several dairy cow studies report increased occurrence of mastitis during the summer [120,121]. Improved survival capability or multiplication of pathogens or their vectors [122] or an adverse action of HS on defensive mechanisms [123] have been indicated as the potential causes of such epidemiological findings. Risk of ketosis; rumen acidosis; liver function impairment; oxidative stress driven gut patho-morphologies; endotoxemia and inflammatory conditions in the gut; modulation of immune status; and secondary disposition to pathogens, constitute various health consequences in response to HS.

## 11. Consequences for Reproductive System

In the preceding literature, we concluded that HS causes partitioning of nutrients towards maintenance and milk yield demands, thus leaving reproductive requirements unfulfilled. Although HS have direct negative effects on reproduction, but decreased nutrients availability and NEBAL during HS also potentially adds towards decline in reproductive efficiency. HS causes high cortisol secretion [44], while studies show that ACTH could block estradiol-induced estrus behavior [124]. Increased corticosteroid secretion [125] because can inhibit gonadotrophins releasing hormone (GnRH), and thus its dependent luteinizing hormone (LH) secretion [126]. Based on literature reporting HS-caused decrease in LH levels, it can be concluded that in summer, the dominant ovarian follicle develops in a low LH resulting in reduced estradiol secretion from the follicle leading to poor exhibition of estrus behavior, and hence, reduced fertility. Roy and Prakash [127] reported lower plasma progesterone and higher prolactin concentration during the estrous cycle in HS bovines. They concluded that PRL and progesterone levels during the summer and winter are directly correlated with the reproductive performance and that hyperprolactinemia may cause acyclicity/infertility during the hot season. Aforementioned endocrine changes having final consequences for reduced reproductive efficiency stems from the homeorehtic responses of dairy cows towards HS [36]. As we mentioned earlier in the case of lactation changes, where a primary resource shift occurs in favor of survivability instead of milk production, the same is true in the case of reproduction. Together with hormone driven reduction of reproduction process; HS also poses direct and indirect consequences for the reproductive system. Direct effects originate from the impairment of the follicular environment of the ovaries and contained oocyte developmental competence [128], while indirect effects stem from HS-driven effects on proteins and hormones vital for their productive system [129]. Other things include serum hypocalcemia, which is implicated in the risk of uterine diseases [130], while HS has also been associated with impaired embryo development and increased embryo mortality in the cattle [131].

## 12. Mitigation Strategies

**Figure 2:**
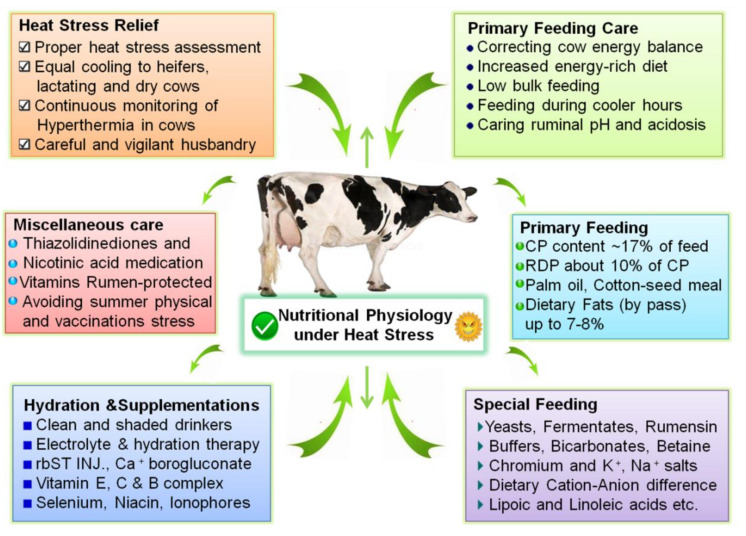
Summary of various impact mitigation strategies to support heat stress-related physiological and biochemical disequilibrium at the various systems-level and improve the production and welfare of dairy cows. (CP=crude protein, RDP=rumen-degradable protein, rbST=recombinant bovine somatotrophins).

Given the huge economic concerns of HS towards dairy production, mitigation strategies could be divided into short- and long-term based. Immediate short- and long-term strategies of mitigation should be based upon cooling measures and improved feeding practices and relevant supplementations. Long-term mitigation approaches are based on proper investment in HS assessment and cooling aids. Genetic selection for high production has reduced heat tolerance among dairy cattle; the identification of heat-tolerant animals within high-producing breeds is very useful through recordings [32] and allied phenotypes [33]. They must be capable of maintaining high production and survival rate under the HS conditions [37]. Besides identification of thermo-tolerant cattle, other long term approach against the HS could be based on differences in anatomical and morphological characteristics, which partially explain differences in heat tolerance among species and breeds [37,101,132,133]. These conditions, including slick hair, white coat color, and low coat density, can be incorporated. Regular HS assessment and applying proper cooling-facilitative measures are very important to the cows; in hot weather. HS cows, when cooled, exhibited stronger NEFA responses to insulin challenge and typical high insulin response of HS explained earlier was reduced [72]. Growth hormones injections, like rbST (recombinant bovine somatotrophins), are shown to increase production in HS cows [4] by increasing NEFA turnover. Injecting rbST is typically known to improve metabolic profile and increase immune response of stressed dairy cows [134]. HS effects are there on non-lactating and dry period cows [72,135] through their subsequent performance; therefore, in the long run, dry period cooling of the cows is also essential. Figure 2 summarizes a score of mitigation strategies to be applied at a dairy operation to relieve the untoward effects of the HS and improve production.

Adapting to feeding ingredients that help to alleviate the negative effects of HS; improving feeding, husbandry practices, and use of minerals and vitamin supplementations; are proven to be helpful. To compensate for the reduced intake caused by HS and high bodily heat of feeding forages, increase the energy-rich rations like extra concentrates. Increasing the energy-density of feed [136] is very important. However, this practice should be conducted with care as this type of diet can be associated with a lower rumen pH and acidosis.

Reduced rumen degradable and un-degradable proteins increases the use of amino acids to maintain milk protein synthesis and limit their catabolism in the cattle exposed to warm climates [137]. Propionate supplementation is useful, as it has been shown to improve metabolism status and milk yield in the transition cows [138]. Propionate is primarily converted into glucose; ratio of its conversion in the rumen is above 30% [139]. Ionophores and monensin feeding is shown to have a positive effect on energetic metabolism and production parameters of cattle under HS [140,141]. Besides crude protein, dietary DCAD (Dietary Cation–Anion Difference) is advised as useful [142]. Dietary bicarbonates (HCO3) could also help significantly due to their buffering properties [143]. Niacin minimizes HS effects and improves metabolism in the lactating dairy cows [144]. Dietary yeast supplementations like, *Aspergillus oryzae* [145] and *Saccharomyces cerevisiae* [146] are the important options available to combat the negative consequences of HS. Dietary CP content of 15.3% and rumen by-pass fats were shown to be beneficial for milk yield maintenance, for cooled cows in summer [147,148]. Palm oil supplementation enhanced the DMI and reduced the HS signs [149]. Feeding of conjugated linoleic acids improved NEBAL during HS, but at the same time, milk fat depression was observed. Lipoic acid has shown to have protective and energetic-metabolism conducive effects [150,151]. Therefore, it is proposed that lipoic acid supplementation could be helpful to the HS animals [152]. A review study has concluded up to 5% of dietary fats to the lactating cows to support energetic metabolism [153]. Trace minerals, like Mn, Zn, Mo, P, and Se are shown to improve metabolic status and improve general health of dairy cows [154]. Vitamins like B-complex, Ascorbic acid, Vitamin E (tocopherol), rumen-protected Niacin and Nicotinic acid [144,155] are evaluated to be beneficial. Thiazolidinediones TZDs can augment HSPs production [156], improve glucose utilization [157] and energetic metabolism [158]; therefore, it could be a useful strategy during HS. Similar to TZDs, dietary betaine could be a better option in heat stressed lactating cows [159]. Chromium supplementation is also being shown to have improved energy metabolism and production in the heat stressed lactating cows [160,161,162].

## 13. Conclusions

It is concluded that the HS acclimation process causes several physiological, endocrinal, and biochemical changes in the dairy cattle. Major reshuffles in energetic metabolism are particularly accountable for dairy production losses under the influence of HS. All these mechanisms ending up in the production losses need to be explored further, in order to generate relevant comprehensive knowledge. Further research is needed about understanding insulin dynamics, especially tissue specific changes under heat stress. The nexus of nutrients absorption, immune responses, and roles of microbiota, constitutes excellent future directions. Identification of quantitative traits loci of genes related to energetic metabolism and production under thermal stress will enhance genomic selection based breeding of cattle with better adaptability. We have a score of mitigation strategies available to provide a certain degree of relief to HS cows and subsequently increase dairy production. Fatty acids based supplementations, plant extracts, novel probiotics, and microbe-based supplementations are the new mitigation avenues, which could be focused on. A fair amount of knowledge is available on this important issue and interesting future discoveries will continue, and so as the new impact mitigation strategies.
